# Mental health symptoms in relation to socio-economic conditions and lifestyle factors – a population-based study in Sweden

**DOI:** 10.1186/1471-2458-9-302

**Published:** 2009-08-20

**Authors:** Anu Molarius, Kenneth Berglund, Charli Eriksson, Hans G Eriksson, Margareta Lindén-Boström, Eva Nordström, Carina Persson, Lotta Sahlqvist, Bengt Starrin, Berit Ydreborg

**Affiliations:** 1 Västmanland County Council, Department of Community Medicine, Västerås, Sweden; 2 Uppsala County Council, Department of Community Medicine, Uppsala, Sweden; 3 Örebro University, Department of Health Sciences, Örebro, Sweden; 4 Sörmland County Council, Department of Community Medicine, Eskilstuna, Sweden; 5 Örebro County Council, Department of Community Medicine, Örebro, Sweden; 6 Karlstad University, Department of Social Studies, Karlstad, Sweden

## Abstract

**Background:**

Poor mental health has large social and economic consequences both for the individual and society. In Sweden, the prevalence of mental health symptoms has increased since the beginning of the 1990s. There is a need for a better understanding of the area for planning preventive activities and health care.

**Methods:**

The study is based on a postal survey questionnaire sent to a random sample of men and women aged 18–84 years in 2004. The overall response rate was 64%. The area investigated covers 55 municipalities with about one million inhabitants in central part of Sweden. The study population includes 42,448 respondents. Mental health was measured with self-reported symptoms of anxiety/depression (EQ-5D, 5th question). The association between socio-economic conditions, lifestyle factors and mental health symptoms was investigated using multivariate multinomial logistic regression models.

**Results:**

About 40% of women and 30% of men reported that they were moderately or extremely anxious or depressed. Younger subjects reported poorer mental health than older subjects, the best mental health was found at ages 65–74 years.

Factors that were strongly and independently related to mental health symptoms were poor social support, experiences of being belittled, employment status (receiving a disability pension and unemployment), economic hardship, critical life events, and functional disability. A strong association was also found between how burdensome domestic work was experienced and anxiety/depression. This was true for both men and women. Educational level was not associated with mental health symptoms.

Of lifestyle factors, physical inactivity, underweight and risk consumption of alcohol were independently associated with mental health symptoms.

**Conclusion:**

Our results support the notion that a ground for good mental health includes balance in social relations, in domestic work and in employment as well as in personal economy both among men and women. In addition, physical inactivity, underweight and risk consumption of alcohol are associated with mental health symptoms independent of socio-economic factors.

## Background

Mental health is an important part of public health. According to the Swedish national public health report [1] between 20 and 40 percent of the general population suffer from poor mental health – everything from severe psychiatric disorders such as psychosis to milder mental health symptoms such as nervousness, anxiety or sleeping problems. Whereas the most severe psychiatric disorders have not increased in the population in Sweden during the last decades, there has been an increase in the prevalence of mental health symptoms since the beginning of the 1990s. Poor mental health has large economic and social consequences both for the individual and society. The costs to society for health care, sickness absence, disability pension and loss of production due to poor mental health were estimated to 50,000 million crowns in Sweden in 1997 [2].

Results from previous studies show strong associations between mental health and e.g. social relations, income, working conditions and critical life events [3-7]. In general, persons with low socio-economic status have poorer mental health than persons with high socio-economic status [8]. Some lifestyle factors, such as physical activity [9,10], alcohol consumption [11,12] and obesity [13] have also been found to be related with mental health. In addition, domestic work has been found to be associated with mental well-being among women [14]. There is a need for a better understanding of these associations in order to plan preventive activities and health care.

The aim of the present study was to estimate the prevalence of self-reported mental health symptoms among men and women in different age groups in the general population and to disentangle the associations between socio-economic conditions, lifestyle factors and mental health symptoms. As a starting point, we used a model of mental health indicators which has been established in a working group in the European Union [3], and which includes e.g. social relations, economic factors, working conditions and critical life events. We extended the model by including domestic work and lifestyle factors in the study.

## Methods

The study is based on a postal survey questionnaire sent to a random sample of men and women aged 18–84 years in autumn 2004. The aim of the survey was to investigate the health status, lifestyle factors and living conditions as well as health care use in the population. The sampling was random at individual level and stratified by gender, age group, county and municipality. The data collection was completed after two postal reminders. The overall response rate was 64 percent. The area investigated covers 55 municipalities in five counties with about one million inhabitants in central part of Sweden. The study population includes 42,448 respondents.

Mental health symptoms were measured with a question about anxiety/depression (EQ-5D, 5th question). EQ-5D [15] is a standardised instrument including five questions that measure health related quality of life. The 5th question represents mental health and is as follows. "Please indicate which statements best describe your own health state today: Anxiety/Depression", with answer options I am not anxious or depressed, I am moderately anxious or depressed and I am extremely anxious or depressed.

### Socio-economic conditions

Educational level was obtained through record linkage to information from a national education register and was categorised into three classes: low (elementary school), medium (upper secondary school), and high (at least 3 years of university or corresponding education). Country of origin was obtained by record linkage to a national population register. The respondents were categorised into those born in Sweden, in other Nordic countries, in other European countries and outside Europe. Family status was obtained from a survey question and categorised into living alone, living with partner, living with partner and children, single parent and other.

Employment status was derived from a survey question about whether the respondent was employed, self-employed, student, on parental leave, unemployed, working at home, on disability pension, retired or other. Economic hardship was assessed by asking whether the respondent had had problems with paying running bills during the last 12 months (no problems; yes, during 1–2 months; yes, during 3–12 months).

Social support was assessed with the question "Do you have any persons in your surrounding you can get support from in case of emotional crises or problems?" with the answer options yes, definitely; yes, probably; probably not and definitely not. The participants were also asked whether they had experienced that someone had belittled them during the last three months. The answer categories were never, once or twice, and several times during the last three months.

There were two questions about domestic work. The first asked how many hours per week the respondent spent working at home that was not paid work (e.g. taking care of children, nursing relatives, buying the groceries, cooking, paying the bills, washing the laundry, cleaning, taking care of a car, house or garden). The second question asked how often the respondent experienced domestic work as burdensome (all or most of the time, sometimes, seldom, never).

The statement: "One can trust the people living in this neighbourhood" was used to evaluate neighbourhood social cohesion where agreement was coded as good, partial disagreement as less good and total disagreement as poor social cohesion. Participation in associations was asked with a question whether the respondent was an active member in an association (trade union, political party, nature/environmental association, sports club, pensioners association, religious association, cultural association, administrative board, other).

Physical environment was derived from a question: "How often do you have disturbance in or around your house from the following sources?" with the alternatives: noise from outside, exhaust from outside, disturbing industry, draught and cold, disturbing neighbours, bad smell, poor quality of drinking water, littered environment, damage or graffiti and other disturbance with the options often, sometimes, seldom and never. Option never was then coded as 0, sometimes or seldom were coded as 1 and often as 2 for each of the disturbances. If the sum was 0–2 the physical environment was coded as good, 3–5 was coded as less good and 6 or more was coded as poor physical environment.

The respondent was defined as functionally disable if she/he needed help on a daily basis due to functional disability or illness. Critical life events during the last two years (death of a near relative, own or a relative's severe illness, separated from a spouse or a partner, being laid off from work, other critical life event) were asked and dichotomised into no or at least one event.

### Lifestyle factors

Physical activity was measured with the question: "How much do you exercise physically in your leisure time?" with the options little exercise (walking, bicycling or other light exercise less than 2 hours a week), moderate exercise (walking, bicycling or other light exercise more than 2 hours a week), moderate regular exercise (exercising 1–2 times a week at least for half an hour at a time in jogging, playing tennis, bicycling, exercising at a gym or other moderate exercise that makes one to sweat) and vigorous exercise and training (exercising or competing at least 3 times a week at least for half an hour at a time in team sports, jogging, playing tennis, swimming or other intensive physical activity). The two middle categories were combined into moderate exercise.

Smoking habits and snuff use were derived from the questionnaire, combined and dichotomised into any cigarette smoking or snuff use daily and not daily. Alcohol consumption was measured using the first three questions in the WHO instrument AUDIT (Alcohol Use Disorders Identification Test). These three questions measure the frequency and quantity of alcohol consumption and relate to risk consumption of alcohol [16].

Relative weight was measured by using body mass index (BMI). BMI was calculated from self-reported weight and height as weight divided with height squared (kg/m^2^). The participants were categorised according to the WHO guidelines [17] as underweight when BMI was lower than 18.5 kg/m^2^, normal weight when BMI was between 18.5 and 24.9 kg/m^2^, overweight when BMI was between 25 and 29.9 kg/m^2^, and obese when BMI was equal to or over 30 kg/m^2^.

The respondents gave their informed consent to use the national register data by answering the questionnaire. The personal identification numbers were deleted directly after the record linkage with the national registers and the survey data are thus anonymous. The survey was approved by the boards of the five county councils and the confidentiality of the data is assured under the Swedish law.

### Statistical analyses

**Figure 1:**
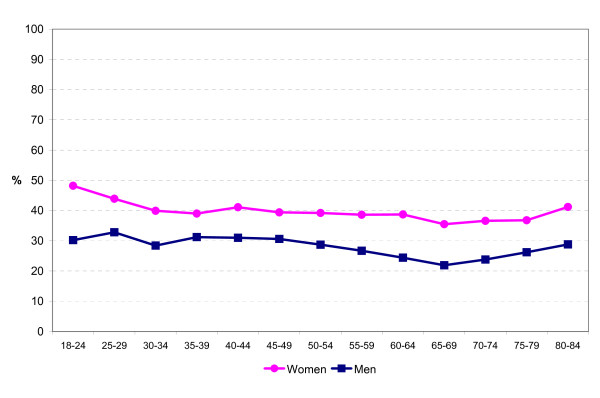
**Prevalence of being extremely or moderately anxious or depressed among women and men in different age groups**.

The prevalence of mental health symptoms (using EQ-5D, 5th question) is reported by gender and age group (Figure 1). The association between socio-economic conditions, lifestyle factors and mental health symptoms was investigated using multinomial logistic regression models. The results are reported as odds ratios (OR) and 95 percent confidence intervals (95% CI) for being extremely or moderately anxious/depressed, respectively, when adjusting for all the other variables in the model (Table 1). The category of not anxious/depressed was the constant category. Since the associations between the studied factors and mental health symptoms were fairly similar in men and women, the combined analyses are reported in this paper, adjusted for gender. Some differences in these associations between men and women are, however, commented in the text. Age was not independently associated with mental health symptoms and was therefore not included in the fully adjusted model (Table 1). The variables that were statistically significantly related to anxiety/depression in univariate analyses were included in the fully adjusted model.

**Table 1:** Odds ratios (with 95% confidence intervals) for being extremely or moderately anxious or depressed.

**Variable**	**Category**	**N**	**OR for extremely anxious/depressed**	**OR for moderately anxious/depressed**
***Socio-economic conditions***				

**Social support**	No	661	5.3 (3.7, 7.4)	1.8 (1.5, 2.1)
	
	Probably not	858	6.6 (4.8, 9.0)	3.0 (2.6, 3.6)
	
	Probably yes	7314	2.3 (1.9, 2.8)	1.6 (1.5, 1.7)
	
	Yes, definitely (ref.)	25812	1	1

**Being belittled (last 3 months)**	Several times	1097	11.5 (8.8, 15.1)	3.6 (3.1, 4.2)
	
	Once or twice	6786	3.3 (2.7, 4.0)	2.1 (2.0, 2.2)
	
	Never (ref.)	26762	1	1

**Employment status**	Disability pensioner	1858	8.5 (6.5, 11.0)	2.9 (2.6, 3.3)
	
	Unemployed	1697	2.9 (2.2, 4.0)	1.6 (1.4, 1.8)
	
	Student	2068	2.0 (1.4, 2.8)	1.3 (1.1, 1.4)
	
	Retired	8516	1.4 (1.1, 1.9)	1.3 (1.2, 1.4)
	
	Self-employed	1953	1.1 (0.7, 1.9)	1.0 (0.9, 1.1)
	
	Parental leave	672	1.1 (0.6, 2.2)	0.8 (0.6, 0.9)
	
	Other	1119	4.3 (3.1, 6.1)	1.5 (1.3, 1.8)
	
	Employed (ref.)	16762	1	1

**Economic hardship (last 12 months)**	Yes, 3–12 months	2042	3.1 (2.4, 3.9)	1.9 (1.7, 2.1)
	
	Yes, 1–2 months	3242	1.5 (1.2, 1.9)	1.4 (1.3, 1.5)
	
	No (ref.)	29361	1	1

**Domestic work burdensome**	All the time	2540	7.2 (5.4, 9.7)	3.0 (2.7, 3.4)
	
	Sometimes	13994	2.3 (1.8, 3.0)	1.9 (1.8, 2.0)
	
	Seldom	8538	1.1 (0.8, 1.5)	1.3 (1.2, 1.4)
	
	Never (ref.)	9573	1	1

**Critical life events (last 2 years)**	Yes	19006	2.2 (1.8, 2.6)	1.4 (1.4, 1.5)
	
	No (ref.)	15639	1	1

**Functional disability**	Yes	1684	3.4 (2.6, 4.4)	2.0 (1.8, 2.3)
	
	No (ref.)	32961	1	1

**Family status**	Living with partner	13845	1.3 (1.0, 1.6)	1.2 (1.1, 1.3)
	
	Living alone	5868	2.1 (1.6, 2.6)	1.4 (1.3, 1.5)
	
	Single parent	2126	1.3 (1.0, 1.7)	1.2 (1.1, 1.3)
	
	Other	1693	1.5 (1.0, 2.2)	1.3 (1.2, 1.5)
	
	Living with partner and children (ref.)	11113	1	1

**Physical environment**	Poor	4081	1.5 (1.2, 1.8)	1.5 (1.4, 1.6)
	
	Less good	10080	1.2 (1.0, 1.5)	1.3 (1.2, 1.3)
	
	Good (ref.)	20484	1	1

**Participation in associations**	No	21386	1.9 (1.5, 2.3)	1.3 (1.3, 1.4)
	
	Yes (ref.)	13259	1	1

**Neighbourhood social cohesion**	Poor	498	1.2 (0.8, 1.9)	1.3 (1.0, 1.6)
	
	Less good	1637	1.4 (1.1, 1.9)	1.2 (1.1, 1.4)
	
	Good (ref.)	32510	1	1

***Background factors***				

**Gender**	Female	18443	1.3 (1.1, 1.6)	1.5 (1.4, 1.6)
	
	Male (ref.)	16202	1	1

**Country of birth**	Outside Europe	1025	2.0 (1.4, 2.8)	1.3 (1.1, 1.5)
	
	Other European country	923	2.0 (1.3, 2.9)	1.3 (1.1, 1.5)
	
	Other Nordic country	1694	0.8 (0.6, 1.2)	1.0 (0.9, 1.2)
	
	Sweden (ref.)	31003	1	1

***Lifestyle factors***				

**Physical activity**	Inactive (<2 h/week)	6070	2.2 (1.6, 3.1)	1.6 (1.4, 1.7)
	
	Moderate	23897	1.4 (1.0, 1.9)	1.3 (1.2, 1.4)
	
	Vigorous (ref.)	4678	1	1

**Smoking or snuff use**	Daily	8346	1.4 (1.2, 1.7)	1.2 (1.1, 1.2)
	
	Not daily (ref.)	26299	1	1

**Risk consumption of alcohol**	Yes	2078	1.6 (1.2, 2.1)	1.4 (1.3, 1.6)
	
	No (ref.)	32567	1	1

**Body mass index**	Underweight (<18.5)	466	2.3 (1.4, 3.6)	1.5 (1.2, 1.9)
	
	Normal weight (ref.)	16727	1	1
	
	Overweight (25–29.9)	12869	0.9 (0.8, 1.1)	1.0 (1.0, 1.1)
	
	Obese (>30)	4583	0.9 (0.7, 1.2)	1.0 (1.0, 1.1)

Multinomial logistic regression, all independent variables are included in the same model, age range 18–84 years.

## Results

About 40 percent of women and 30 percent of men reported that they were moderately or extremely anxious or depressed. The prevalence of mental health symptoms was higher among younger than older subjects. The best mental health was found at ages 65–74 years (Figure 1).

Of the 34 645 subjects aged 18–84 years who answered all the questions included in the multiple multinomial logistic regression analysis, 10 697 (31 percent) reported that they were moderately anxious or depressed, whereas 672 (2 percent) reported that they were extremely anxious or depressed.

Factors that were strongly and independently related with anxiety/depression were poor social support, experiences of being belittled, employment status (receiving a disability pension and unemployment), economic hardship, critical life events, and functional disability (Table 1). There was no association between the number of hours spent in domestic work and mental health symptoms. Instead a strong independent association was found between how burdensome domestic work was experienced and anxiety/depression. This was true for both men and women.

Country of origin and family status were also associated with mental health symptoms (Table 1). Subjects born in other European countries and outside Europe were more often anxious or depressed than those born in Nordic countries. Persons living alone had a higher prevalence of anxiety/depression than persons living with partner and children. Single parents had a high odds ratio for being extremely anxious/depressed in the univariate analysis (OR: 3.5, 95% CI: 2.8, 4.4) but this association attenuated considerably when adjusting for socio-economic and lifestyle factors.

Participation in associations, neighbourhood social cohesion and physical environment were only slightly associated with mental health symptoms when adjusted for the other factors included in the model. Educational level and age were not independently associated with mental health symptoms.

Of lifestyle factors, physical inactivity, underweight and risk consumption of alcohol were independently associated with anxiety/depression. Underweight was associated with anxiety/depression especially among women and risk consumption of alcohol especially among men (not shown).

## Discussion

Whereas the most severe psychiatric disorders, such as psychoses, have not increased in the population in Sweden during the last decades, there has been an increase in the prevalence of nervousness and anxiety since the beginning of the 1990s [1]. One possible explanation that has been mentioned is that it has become more socially accepted to tell about nervousness or anxiety. The increased premature mortality and psychiatric morbidity associated with these symptoms has, however, been relatively stable during the last ten years, indicating that self-reported severe mental health symptoms are good indicators of psychiatric morbidity [18].

Our results show that women report mental health symptoms to a larger extent than men do. It is plausible that this has to do with the position of women in society. Even though there is a relatively high equality of opportunities between genders in Sweden, women still have a high workload both at work and at home [19] and therefore also a higher level of stress hormones [20].

Young adults have a higher prevalence of mental health symptoms than older subjects do. Nearly half of women and one third of men aged 18–34 years reported that they were moderately or extremely anxious or depressed. The prevalence of mental health symptoms decreased with age until the age of 70–74 years and increased again among those over 75 years. Many factors that have been shown to be associated with mental health symptoms in the present and other studies (such as unemployment, economic hardship and being belittled) are more prevalent among younger than older subjects.

### Factors associated with mental health symptoms

Social relations are in many ways important for mental health [4]. Social support is a protecting factor that acts a buffer in psychosocial crisis situations and strain [21]. Poor social support and being belittled were strongly related with mental health symptoms in the present study. Previous studies indicate that experiences of shame are associated with poor mental health for example among the unemployed [22].

Personal economy had also a strong association with mental health symptoms. Subjects with economic problems had a higher prevalence of anxiety/depression than subjects without economic problems. Previous studies have indicated that economic hardship both at present [5,23,24] and under childhood [24] is strongly associated with poor mental health.

There was no association between the number of hours spent in domestic work such as taking care of children, nursing relatives, buying the groceries, cooking, washing the laundry, cleaning etc. and mental health symptoms. Instead a strong independent association was found between how burdensome domestic work was experienced and anxiety/depression. Subjects who often or all the time experienced domestic work as burdensome had an increased prevalence of mental health symptoms. This was true as well for women as for men. Previous studies have reported domestic work as a risk factor for poor health among women, particularly in combination with work-related stress [14,20,25], whereas the association has been less often studied or found weaker among men.

Critical life events, such as death of a near relative, own or a relative's severe illness, separation from a spouse or a partner or being laid off from work, were associated with mental health symptoms in the present study. These events can be a triggering factor for poor mental health because they require a high level of psychological adaptation [21]. There is also an association between physical ill health and mental ill health [1,3]. In the present study, a factor that was strongly related to mental health symptoms was functional disability i.e. being dependent on help from others to manage everyday life.

Single parents have been found to have higher level of mental health problems than population in general [1,26]. In the present study, there was a strong crude association between being single parent and mental health symptoms. This association, however, almost disappeared when adjusted for other socio-economic conditions and lifestyle factors, suggesting that the increased level of anxiety/depression among single parents can be explained by these factors. For example, burdensome domestic work and economic hardship are more prevalent among single parents than parents living together. This should be taken into consideration when reporting differences in mental health symptoms between different family constellations. On the contrary, the association between living alone and anxiety/depression remained about the same even after the adjustment.

There was also an association between country of origin and mental health symptoms. Subjects born in other European countries and outside Europe were more often anxious or depressed than those born in Nordic countries which is in line with previous studies [1]. Women had a somewhat higher prevalence of anxiety/depression than men even when socio-economic conditions and lifestyle factors were taken into account.

Working conditions, such as high demands in combination with low control at work and job insecurity have been shown to be detrimental for health [6,7,27]. To elucidate the role of working conditions was, however, beyond the scope of the present study. Subjects who were not employed, such as disability pensioners and the unemployed, had a higher level of anxiety/depression than the employed, which is in agreement with previous studies [1,7,28].

Physical inactivity was associated with mental health symptoms in the present study. This is in line with previous studies where physical activity has been shown to have a positive effect on mental health [9,10]. Underweight subjects had a higher prevalence of mental health symptoms than normal weight subjects, especially among women, corroborating previous studies [29]. Underweight can be an effect of an eating disorder, which in turn is related to poor mental health. Contrary to previous research [13] there was, however, no association between obesity and anxiety/depression when adjusted for socio-economic and other lifestyle factors.

A high and long lasting consumption of alcohol increases the risk of alcohol related injuries, suicide, depression and anxiety [11,12]. It has been shown in national studies in Sweden that risk consumption of alcohol is related with depression and anxiety [11]. In the present study, there was a strong independent association between risk consumption of alcohol and self-reported anxiety/depression among men.

### Strengths and limitations of the study

Since the present study is based on cross-sectional data, it is not possible to say which are causes and which are effects of mental health symptoms. In many cases the relationships are bi-directional [3,30]. For example, problems in social relations can lead to mental health symptoms, but poor mental health can also lead to problems in social relations. Economic hardship can cause anxiety or depression, but anxiety/depression can lead to economic hardship through lower income due to sickness absence or disability pension. Furthermore, burdensome domestic work can lead to mental health symptoms, but poor mental health can also lead to that one experiences domestic work as burdensome.

The response rate of the present study was 64 percent. The response rate was lower among younger than older subjects and among men compared with women. The level of education was also somewhat higher among the respondents than among the general population of the same age. Those who suffer from severe psychiatric disorders are probably underrepresented. Therefore the absolute levels of self-reported mental health symptoms should be interpreted with caution. It is, however, unlikely that the associations between mental health symptoms and other factors reported in the present study could have been explained by non-response.

A strength of the present study is that it is large and population-based. It comprises a study population of over 42,000 individuals and represents about one million inhabitants aged 18–84 years in Sweden. We could even study factors that are rare in the general population and take into account a wide range of socio-economic and lifestyle factors at the same time.

EQ-5D is an internationally validated scale of quality of life where the fifth dimension measures anxiety/depression [15,31]. Another widely used measure of mental health is GHQ-12, the twelve-item version of the General Health Questionnaire [32], which was also measured in the present study. We used EQ-5D to analyse the association between the studied socio-economic and lifestyle factors and mental health because it gives more information about the severity of mental health symptoms than using one cut-off point for GHQ-12. The results were, however, similar when using GHQ-12 instead of EQ-5D as the dependent variable, which gives further support to the findings of the study.

As a starting point of the study, we used a model of mental health indicators which has been established in a working group in the European Union [3]. It includes e.g. social relations, economic factors, working conditions and critical life events. We were, however, able to extend the model by elucidating the importance of domestic work and lifestyle factors in the same context.

## Conclusion

Our results support the notion that a ground for good mental health includes balance in social relations, in domestic work, in employment as well as in personal economy. This is in line with previous studies, but adds domestic work as one of the key factors both among men and women. In addition, lifestyle factors such as physical inactivity, underweight and risk consumption of alcohol seem to be associated with mental health symptoms independent of socio-economic factors. It would be valuable to take into account all these areas of life when planning activities to prevent mental health symptoms, highly prevalent in the general population, and when promoting mental health. Furthermore, an individual will be able to better handle psychosocial crisis situations or strain if she/he possesses a wide array of protecting factors.

## Competing interests

The authors declare that they have no competing interests.

## Authors' contributions

All authors participated in acquisition of the data, design of the study and helped to draft the manuscript. AM co-ordinated the study and drafted the manuscript. HE, CP, KB and MLB performed the statistical analyses. All authors read and approved the final manuscript.

## Pre-publication history

The pre-publication history for this paper can be accessed here:


